# Laparoscopic Surgery for Colovesical Fistula Caused by Colonic Diverticulitis: A Retrospective Study at a Single Center

**DOI:** 10.7759/cureus.82810

**Published:** 2025-04-22

**Authors:** Kazuki Kawasaki, Tomohiro Kurokawa, Tetuya Kurosaki

**Affiliations:** 1 Surgery, Itabashi Chuo Medical Center, Tokyo, JPN; 2 Surgery, Jyoban Hospital, Iwaki, JPN; 3 Surgery, San-ai Hospital, Saitama, JPN

**Keywords:** colovesical fistula, diverticulitis, laparoscopic surgery, minimally invasive surgery, sigmoid colon

## Abstract

Background

Colovesical fistulas caused by colonic diverticulitis are relatively rare but require surgical intervention. Recent advancements have enabled laparoscopic approaches, although standard procedures are yet to be established.

Objective

To evaluate the safety and efficacy of laparoscopic surgery for colovesical fistula and compare outcomes with those for Hinchey I/II diverticulitis without fistulas.

Methods

We retrospectively reviewed 48 patients who underwent laparoscopic surgery for colonic diverticulitis from January 2015 to August 2024. Of these, nine had colovesical fistulas. Operative time, blood loss, and postoperative hospital stay were compared using Welch’s t-test.

Results

The mean operative time was 381.2 ± 175.0 min in the fistula group and 308.1 ± 120.5 min in the non-fistula group (p = 0.262). Mean blood loss was 162.8 ± 167.4 mL and 130.8 ± 197.7 mL, respectively (p = 0.626). Mean postoperative hospital stay was 7.7 ± 1.12 days and 7.4 ± 2.27 days (p = 0.626), respectively. There were no conversions to open surgery, and no recurrences or severe complications were observed.

Conclusion

Laparoscopic surgery for colovesical fistula is feasible and achieves outcomes comparable to standard cases of Hinchey I/II diverticulitis. A standardized "all-in-one" laparoscopic technique can be applied even in cases with fistulas.

## Introduction

In Japan, the incidence of left-sided colonic diverticulitis has increased due to the Westernization of dietary habits [1]. According to previous large-scale studies, approximately 2-4% of patients with colonic diverticulitis develop colovesical fistulas, most commonly involving the sigmoid colon [2,3]. Surgery remains the mainstay of treatment. While open surgery was traditionally performed, the global trend has shifted toward minimally invasive approaches [4,5,6,7], with laparoscopic surgery now considered the preferred method due to its favorable outcomes. We have adopted a proactive laparoscopic approach for recurrent diverticulitis and cases with colovesical fistulas. This study evaluates our surgical strategies and compares outcomes between patients with and without fistulas to determine whether a standardized 'all-in-one' laparoscopic approach is applicable.

## Materials and methods

Study design and setting

This retrospective observational study was conducted at the Itabashi Chuo Medical Center, a community-based core medical institution in Tokyo, Japan, between January 2015 and August 2024.

Inclusion and exclusion criteria

Inclusion criteria were: (1) patients aged 18 years or older, (2) diagnosis of Hinchey stage I or II diverticulitis, and (3) laparoscopic surgery performed at our institution. Patients were included in the fistula group if a colovesical fistula was identified intraoperatively.

Exclusion criteria were: (1) patients with Hinchey stage III or IV diverticulitis, (2) suspected or confirmed colorectal malignancy, and (3) cases initially managed with open surgery or converted from laparoscopic to open approach.

In this study, recurrent diverticulitis was defined as two or more documented episodes of acute diverticulitis requiring medical treatment. Surgical indications were based on the Japanese guidelines, which recommend elective surgery after multiple recurrences to prevent complications and improve quality of life.

Sampling technique

All eligible patients meeting the criteria were consecutively enrolled.

Ethical considerations

This study was approved by the Ethics Committee of Itabashi Chuo Medical Center (Approval Number: 230725B). Given the retrospective nature of the study, the requirement for written informed consent was waived. An opt-out method was used in accordance with institutional guidelines, allowing patients the opportunity to decline participation.

Preoperative evaluation

CT imaging demonstrated wall thickening of the colon and bladder, abscess formation, and low-density tracts suggestive of fistulas. In cases with pneumaturia, cystoscopy and MRI were selectively employed to confirm the fistula location and rule out malignancy. Colonoscopy was performed unless contraindicated.

All 48 patients underwent preoperative CT to evaluate colonic wall thickening, pericolic inflammation, abscess formation, and suspected fistulous tracts. MRI was selectively performed in nine patients with clinical findings suggestive of colovesical fistulas, such as pneumaturia or bladder wall thickening observed on CT. According to recent literature, CT demonstrates a sensitivity of approximately 60-80% for detecting colovesical fistulas, while MRI has a higher sensitivity, ranging from 80-100%, and it better delineates fistulous tracts and bladder involvement [8].

Surgical technique

All procedures were performed laparoscopically. Each operation was supervised by an experienced colorectal surgeon, who participated either as the primary operator or as an assistant. The patient was placed in the lithotomy position, and pneumoperitoneum was established using a 12-mm umbilical trocar at the umbilicus, followed by the insertion of four to five additional ports. The surgical approach consistently involved complete mobilization of the splenic flexure to ensure a tension-free anastomosis. Dissection was initiated using a lateral-to-medial approach. Since lymph node dissection is not required in benign diverticular disease, only minimal mesenteric mobilization was performed. The mesentery was dissected close to the bowel wall to avoid unnecessary tissue damage and preserve surrounding structures. This approach allows for adequate resection length while minimizing trauma. Additionally, because rectal involvement is typically minimal in these cases, bowel transection was performed with the intention of preserving an adequate length of the rectum to facilitate safe anastomosis using the double-stapling technique (DST). When DST was not feasible, a functional end-to-end or an overlap anastomosis was performed. In cases where the overlap technique was used, the anastomosis was performed in an intracorporeal fashion. In cases with colovesical fistulas, the bladder was carefully inspected, and any visible defect was closed primarily with interrupted or running absorbable sutures. Methylene blue was instilled into the bladder to confirm the integrity of the repair when necessary. A drain was placed selectively based on intraoperative findings.

Statistical analysis

Continuous variables are presented as means ± standard deviations and were compared using Welch’s t-test. A p-value < 0.05 was considered statistically significant. Analyses were performed using EZR (Saitama Medical Center, Jichi Medical University, Japan).

No formal power calculation was performed due to the retrospective nature of this study and the limited number of patients in the fistula group. Therefore, the results should be interpreted with caution, particularly given the small sample size.

## Results

All surgeries were completed laparoscopically without conversion to open surgery, and no patients required a diverting stoma. The overall mean operative time was 322 min, the mean blood loss was 137 mL, and the mean postoperative hospital stay was 7.5 days.

In the 39 patients without colovesical fistulas, the mean operative time was 308.1 ± 120.5 min, the mean blood loss was 130.8 ± 197.7 mL, and the mean postoperative stay was 7.4 ± 2.27 days. In the nine fistula cases, the corresponding values were 381.2 ± 175.0 min, 162.8 ± 167.4 mL, and 7.7 ± 1.12 days. Welch’s t-test revealed no significant differences between the two groups (p = 0.262, 0.626, and 0.626, respectively).

Of the 48 patients, 10 had a right-sided colonic diverticulitis and 38 had left-sided disease. All nine cases with colovesical fistulas were associated with left-sided diverticulitis involving the sigmoid colon. Table 1 presents detailed surgical characteristics of the fistula group, including operative time, blood loss, splenic flexure mobilization, and bladder suturing. Splenic flexure mobilization was performed in all nine cases (100%), and primary bladder repair was conducted in five of nine cases (55.6%) where fistulous defects were visible.

**Table 1:** Clinical Data of Patients with Colovesical Fistula (n = 9)

No.	Age	Sex	Operative Time (min)	Blood Loss (mL)	Splenic Flexure Mobilization	Bladder Suturing	Postoperative Hospital Stay (days)
1	40	Male	233	0	No	Yes	6
2	57	Male	707	350	Yes	No	9
3	76	Female	237	50	Yes	No	8
4	49	Male	298	185	No	No	6
5	76	Male	263	30	Yes	Yes	8
6	63	Male	409	300	Yes	Yes	8
7	37	Male	631	450	Yes	No	8
8	64	Male	271	0	Yes	Yes	7
9	42	Male	382	100	Yes	Yes	9

No postoperative recurrences were observed during the follow-up period. One patient in the non-fistula group developed an anastomotic leak, which was successfully managed with conservative treatment.

## Discussion

Diverticulitis remains the most common cause of colovesical fistulas, especially in older populations. While clinical signs such as pneumaturia and fecaluria are suggestive, radiologic modalities such as CT and MRI play a crucial role in confirming fistulas and assessing their anatomy [8,9]. However, confirmation by cystoscopy or contrast studies may not always be feasible.

Recent studies increasingly support laparoscopic surgery as a safe and effective alternative to open procedures [4-7]. Nevertheless, the lack of a universally accepted surgical approach is due to the technical complexity of managing dense adhesions and bladder wall involvement. Our findings align with those of Tomizawa et al. [1], demonstrating that with careful preoperative planning and intraoperative strategy, laparoscopic management is feasible and safe even in complicated cases.

Several other studies have reported favorable outcomes with laparoscopic repair. Campobasso et al. [10] found that laparoscopic surgery was associated with shorter hospital stays and fewer infections compared to open surgery. In a larger study, Tomizawa et al. [1] emphasized that laparoscopic management offers improved recovery when performed by experienced surgeons. These findings are consistent with our experience and further validate the use of a standardized, reproducible laparoscopic approach.

In accordance with the Japanese guidelines for colonic diverticular disease, elective surgery was indicated for patients with Hinchey I or II diverticulitis who had experienced multiple recurrent episodes, failed conservative treatment, or developed complications such as colovesical fistulas. Several international studies also support surgical intervention in select cases to prevent recurrence and improve quality of life [2,4].

All patients included in this study had a history of recurrent diverticulitis that was refractory to medical treatment, including bowel rest and intravenous antibiotics. According to Japanese diverticulitis guidelines, elective surgery is considered in patients with multiple recurrences or complications such as fistula formation. Therefore, surgical intervention was indicated in these cases to prevent further deterioration and improve quality of life.

Surgical indications for Hinchey I/II diverticulitis were based on the Japanese Guidelines for the Management of Colonic Diverticular Disease (2020 edition), which recommend elective resection after multiple recurrences or complications. This approach aligns with international practices, including European Society of Coloproctology (ESCP) guidelines, which acknowledge the role of surgery in patients with recurrent or complicated diverticulitis when quality of life is impaired or when complications such as fistula arise [4].

Our “all-in-one” technique - featuring complete splenic flexure mobilization, consistent dissection strategy, and selective bladder repair - may help reduce complications such as leakage and recurrence. The absence of conversions or stoma creation in our cohort supports its reliability (Table 2).

**Table 2:** Our “all-in-one” technique

Step	Description
1	Full mobilization of the splenic flexure to ensure tension-free anastomosis
2	Lateral-to-medial dissection of the colon
3	Minimal mesenteric mobilization close to the bowel wall
4	Transection of bowel using the double-stapling technique (DST) or overlap anastomosis
5	Intracorporeal anastomosis when DST not feasible
6	In fistula cases, bladder inspection and repair with absorbable sutures
7	Methylene blue test to confirm bladder repair integrity (when needed)

This study is limited by its retrospective design and small sample size from a single center. However, comparative analysis with non-fistula cases strengthens our conclusion that fistula presence does not increase surgical risk when handled laparoscopically. Prospective, multicenter trials are needed to confirm these findings and improve surgical protocols.

In addition to the retrospective nature and small sample size, this study is also limited by the lack of long-term follow-up and the absence of objective assessment of functional outcomes such as urinary and bowel function. Future prospective studies with longer observation periods and functional evaluation are needed to validate our findings.

## Conclusions

Laparoscopic surgery for colovesical fistulas due to diverticulitis is safe and feasible, producing outcomes comparable to those without fistulas. Our standardized “all-in-one” approach offers a reliable and minimally invasive treatment option that may be broadly implemented. Our laparoscopic approach resulted in no conversions to open surgery and no postoperative recurrences. The clinical outcomes, including operative time, blood loss, and length of stay, were comparable between patients with and without colovesical fistulas.
